# Supplementary material for: Community interventions in Low—And Middle-Income Countries to inform COVID-19 control implementation decisions in Kenya: A rapid systematic review
Source: PLoS One. 2020 Dec 8;15(12):e0242403. doi: 10.1371/journal.pone.0242403 (PMC7723273; doi:10.1371/journal.pone.0242403)
Supplement: S2 Table — shows the detailed search term used in PubMed in the study. (DOCX) [file pone.0242403.s002.docx]

**S2 Table: Search term in Pubmed**

| Community measures and control | distancing OR distance OR social network* OR isolation OR social contact* OR face masks OR hand washing OR hand hygiene OR hand clean, and hand decontaminate OR prevention OR control OR ‘non-pharmaceutical measures’ OR ‘non-pharmaceutical  interventions’ OR ‘social distancing OR school closures OR workplace closures OR mass  gatherings OR hygiene |
| --- | --- |
| Infectious disease outbreak | “pandemics” OR “pandemic” OR outbreak OR “influenza, human” OR “influenza” AND “human influenza” OR “influenza” OR “coronavirus” OR “coronavirus” OR 2019 novel coronavirus  and COVID-19” OR “covid-19” OR “novel coronavirus” OR other coronaviruses OR SARS OR “severe acute respiratory syndrome” OR “severe acute respiratory syndrome” OR “Middle East Respiratory Syndrome Coronavirus” OR “middle east respiratory  syndrome*” OR “MERS-CoV” OR Mers OR  “Middle Eastern Respiratory Syndrome*” OR  “MERSCoV*” OR coronavirus OR Coronavirus  Infections OR coronavirus* OR “COVID-19”  or “2019-nCoV” or “SARS-CoV-2” OR Bovine  Spongiform Encephalitis 1986 OR Avian flu 1997 OR  SARS 2002 OR Swine Flu 2009 OR Ebola 2014 |
| LMIC | Afghanistan OR Albania OR Algeria OR American Samoa OR Angola OR Argentina OR Armenia OR Azerbaijan OR Bangladesh OR Belarus OR Belize OR Benin OR Bhutan OR Bolivia OR Bosnia And Herzegovina OR Botswana OR Brazil OR Bulgaria OR Burkina Faso OR Burundi OR Cabo Verde OR Cambodia OR Cameroon OR Central African Republic OR Chad OR China OR Colombia OR Comoros OR Congo, Dem. Rep. OR Congo, Rep. OR Costa Rica OR Cote D'ivoire OR Cuba OR Djibouti OR Dominica OR Dominican Republic OR Ecuador OR Egypt, Arab Rep. OR El Salvador OR Equatorial Guinea OR Eritrea OR Eswatini OR Ethiopia OR Fiji OR Gabon OR Gambia, The OR Georgia OR Ghana OR Grenada OR Guatemala OR Guinea OR Guinea-Bissau OR Guyana OR Haiti OR Honduras OR India OR Indonesia OR Iran, Islamic Rep. OR Iraq OR Jamaica OR Jordan OR Kazakhstan OR Kenya OR Kiribati OR Korea, Dem. People’s Rep. OR Kosovo OR Kyrgyz Republic OR Lao Pdr OR Lebanon OR Lesotho OR Liberia OR Libya OR Madagascar OR Malawi OR Malaysia OR Maldives OR Mali OR Marshall Islands OR Mauritania OR Mauritius OR Mexico OR Micronesia, Fed. Sts. OR Moldova OR Mongolia OR Montenegro OR Morocco OR Mozambique OR Myanmar OR Namibia OR Nauru OR Nepal OR Nicaragua OR Niger OR Nigeria OR North Macedonia OR Pakistan OR Papua New Guinea OR Paraguay OR Peru OR Philippines OR Romania OR Russian Federation OR Rwanda OR Samoa OR Sao Tome And Principe OR Senegal OR Serbia OR Sierra Leone OR Solomon Islands OR Somalia OR South Africa OR South Sudan OR Sri Lanka OR St. Lucia OR St. Vincent And The Grenadines OR Sudan OR Suriname OR Syrian Arab Republic OR Tajikistan OR Tanzania OR Thailand OR Timor-Leste OR Togo OR Tonga OR Tunisia OR Turkey OR Turkmenistan OR Tuvalu OR Uganda OR Ukraine OR Uzbekistan OR Vanuatu OR Venezuela, Rb OR Vietnam OR West Bank And Gaza OR Yemen, Rep. OR Zambia OR Zimbabwe |
| Combined term | ((((distancing OR distance OR social network* OR isolation OR social contact* OR face masks OR hand washing OR hand hygiene OR hand clean, and hand decontaminate OR prevention OR control))) AND ((“pandemics” OR “pandemic” OR outbreak OR “influenza, human” OR “influenza” AND “human influenza” OR “influenza” OR “coronavirus” OR “coronavirus” OR 2019 novel coronavirus and COVID-19” OR “covid-19” OR “novel coronavirus” OR other coronaviruses OR SARS OR “severe acute respiratory syndrome” OR “severe acute respiratory syndrome” OR “Middle East Respiratory Syndrome Coronavirus” OR “middle east respiratory syndrome*” OR “MERS-CoV” OR Mers OR “Middle Eastern Respiratory Syndrome*” OR “MERSCoV*” OR coronavirus OR Coronavirus Infections OR coronavirus* OR “COVID-19” or “2019-nCoV” or “SARS-CoV-2” OR Bovine Spongiform Encephalitis 1986 OR Avian flu 1997 OR SARS 2002 OR Swine Flu 2009 OR Ebola 2014))) AND ((Afghanistan OR Albania OR Algeria OR American Samoa OR Angola OR Argentina OR Armenia OR Azerbaijan OR Bangladesh OR Belarus OR Belize OR Benin OR Bhutan OR Bolivia OR Bosnia And Herzegovina OR Botswana OR Brazil OR Bulgaria OR Burkina Faso OR Burundi OR Cabo Verde OR Cambodia OR Cameroon OR Central African Republic OR Chad OR China OR Colombia OR Comoros OR Congo, Dem. Rep. OR Congo, Rep. OR Costa Rica OR Cote D'ivoire OR Cuba OR Djibouti OR Dominica OR Dominican Republic OR Ecuador OR Egypt, Arab Rep. OR El Salvador OR Equatorial Guinea OR Eritrea OR Eswatini OR Ethiopia OR Fiji OR Gabon OR Gambia, The OR Georgia OR Ghana OR Grenada OR Guatemala OR Guinea OR Guinea-Bissau OR Guyana OR Haiti OR Honduras OR India OR Indonesia OR Iran, Islamic Rep. OR Iraq OR Jamaica OR Jordan OR Kazakhstan OR Kenya OR Kiribati OR Korea, Dem. People’s Rep. OR Kosovo OR Kyrgyz Republic OR Lao Pdr OR Lebanon OR Lesotho OR Liberia OR Libya OR Madagascar OR Malawi OR Malaysia OR Maldives OR Mali OR Marshall Islands OR Mauritania OR Mauritius OR Mexico OR Micronesia, Fed. Sts. OR Moldova OR Mongolia OR Montenegro OR Morocco OR Mozambique OR Myanmar OR Namibia OR Nauru OR Nepal OR Nicaragua OR Niger OR Nigeria OR North Macedonia OR Pakistan OR Papua New Guinea OR Paraguay OR Peru OR Philippines OR Romania OR Russian Federation OR Rwanda OR Samoa OR Sao Tome And Principe OR Senegal OR Serbia OR Sierra Leone OR Solomon Islands OR Somalia OR South Africa OR South Sudan OR Sri Lanka OR St. Lucia OR St. Vincent And The Grenadines OR Sudan OR Suriname OR Syrian Arab Republic OR Tajikistan OR Tanzania OR Thailand OR TimOR-Leste OR Togo OR Tonga OR Tunisia OR Turkey OR Turkmenistan OR Tuvalu OR Uganda OR Ukraine OR Uzbekistan OR Vanuatu OR Venezuela, Rb OR Vietnam OR West Bank And Gaza OR Yemen, Rep. OR Zambia OR Zimbabwe)) |
