# Supplementary material for: Community interventions in Low—And Middle-Income Countries to inform COVID-19 control implementation decisions in Kenya: A rapid systematic review
Source: PLoS One. 2020 Dec 8;15(12):e0242403. doi: 10.1371/journal.pone.0242403 (PMC7723273; doi:10.1371/journal.pone.0242403)
Supplement: S3 Table — shows the data extraction form template used in the study. (DOCX) [file pone.0242403.s003.docx]

**S3 Table: Data extraction form**

1. **Data description**

| Name of person extracting |  |
| --- | --- |
| Study ID |  |
| Reference citation |  |
| Study author contact details |  |
| Publication type |  |
| Potential references |  |
| Notes |  |

1. **Study characteristics**

| **Key domains** | **Description** | **Page no.** |
| --- | --- | --- |
| Community intervention |  |  |
| Disease targeted |  |  |
| Study design |  |  |
| Participants |  |  |
| Outcome measured |  |  |
| Include or exclude |  | |
| Reason for exclusion |  | |

1. **Methods**

| **Key domains** | **Description** | **Page no.** |
| --- | --- | --- |
| Aim of study |  |  |
| Unit of analysis |  |  |
| Start date |  |  |
| End date |  |  |
| Duration of community intervention |  |  |
| Informed consent obtained |  |  |
| Ethical approval |  |  |
| Study characteristics |  |  |
| Population description |  |  |
| Setting (Location and social context) |  |  |
| Inclusion criteria |  |  |
| Exclusion criteria |  |  |
| Method of recruitment of participants (e.g. phone, mail, clinic patients) |  |  |
| Total no. enrolled |  |  |
| Age |  |  |
| Sex |  |  |

1. **Outcome measured**

| **Key domains** | **Description** | **Page no.** |
| --- | --- | --- |
| Disease |  |  |
| Country |  |  |
| Best practices and innovative community measures |  |  |
| Implementation strategy |  |  |
| Successful completion of outbreak |  |  |
| Management of outbreak |  |  |
| Coping mechanism |  |  |
| Community intervention |  |  |
| Key conclusions from the authors |  |  |
| Notes |  |  |

| 1. **Risk of bias (Quantitative study)**  \| **Key domains** \| **Description** \| **Page no.** \| \| --- \| --- \| --- \| \| Selection bias (allocation concealment, allocation sequence ) \|  \|  \| \| Attrition bias? \|  \|  \| \| Reporting bias? \|  \|  \| \| Are study results valid? \|  \|  \| \| Others (specify) \|  \|  \| |  |  |  |  | Notes |
| --- | --- | --- | --- | --- | --- | --- | --- | --- | --- | --- | --- | --- | --- | --- | --- | --- | --- | --- | --- | --- | --- | --- | --- |

1. **Risk of bias (Qualitative study)**

| **Key domains** | **Description** | **Page no.** |
| --- | --- | --- |
| Is a qualitative approach appropriate? |  |  |
| Is the study clear in what it seeks to do? |  |  |
| How defensible/rigorous is the research design/methodology? |  |  |
| How clear and coherent is the reporting of ethics? |  |  |
| How well was the data collection carried out? |  |  |
| Is the role of the researcher clearly described? |  |  |
| Is the context clearly described? |  |  |
| Were the methods reliable? |  |  |
| Is the data analysis sufficiently rigorous? |  |  |
| Is the analysis reliable? |  |  |
| Are the findings convincing? |  |  |
| Are the findings relevant to the aims of the study? |  |  |
| Is the data 'rich'? |  |  |
| Key conclusions from the authors |  |  |
