# Supplementary material for: Community interventions in Low—And Middle-Income Countries to inform COVID-19 control implementation decisions in Kenya: A rapid systematic review
Source: PLoS One. 2020 Dec 8;15(12):e0242403. doi: 10.1371/journal.pone.0242403 (PMC7723273; doi:10.1371/journal.pone.0242403)
Supplement: S1 File — (DOCX) [file pone.0242403.s004.docx]

**Minimum data used in the analysis**

| **1. Face mask intervention** | |  |  |  |
| --- | --- | --- | --- | --- |
| **Studies** | **Face mask** | | **No face mask** | |
|  | **n** | **N** | **n** | **N** |
| **SARS** |  |  |  |  |
| **Wu 2004** | **93** | **330** | **388** | **660** |
| **Lau 2004** | **27** | **94** | **43** | **281** |
|  |  |  |  |  |
| **Influenza** |  |  |  |  |
| **Zhang 2013** | **0** | **15** | **9** | **26** |
| **Cowling 2009** | **18** | **258** | **28** | **279** |
| **Cowling 2008** | **4** | **61** | **12** | **205** |
|  |  |  |  |  |
| **2. Hand hygiene practices interventions** | | |  |  |
|  | **Hand hygiene** | | **No hand hygiene** | |
| **SARS** |  |  |  |  |
| **Lau 2004** | **61** | **330** | **222** | **660** |
| **Wu 2004** | **78** | **94** | **90** | **281** |
|  |  |  |  |  |
| **Influenza** |  |  |  |  |
| **Cowling 2008** | **7** | **84** | **12** | **205** |
| **Doshi 2015** | **12** | **145** | **12** | **341** |
| **Ram 2015** | **24** | **193** | **36** | **184** |
| **Simmerman 2011** | **66** | **292** | **58** | **302** |
|  |  |  |  |  |
| **3. Multi-component intervention** | | |  |  |
| **a) Combined facemask & hand hygiene vs hand hygiene only** | | | |  |
|  | **Face mask & hand hygiene** | | **Hand hygiene only** | |
| **Cowling 2009** | **6** | **258** | **7** | **257** |
| **Simmerman 2011** | **51** | **191** | **48** | **200** |
|  |  |  |  |  |
| **a) Combined facemask & hand hygiene vs control** | | |  |  |
|  | **Face mask & hand hygiene** | | **No intervention** | |
| **Cowling 2009** | **18** | **258** | **28** | **279** |
| **Simmerman 2011** | **51** | **191** | **45** | **195** |
