# Supplementary material for: Community interventions in Low—And Middle-Income Countries to inform COVID-19 control implementation decisions in Kenya: A rapid systematic review
Source: PLoS One. 2020 Dec 8;15(12):e0242403. doi: 10.1371/journal.pone.0242403 (PMC7723273; doi:10.1371/journal.pone.0242403)
Supplement: S2 File — shows the Summary of Finding table on the quality of evidence on the included 17 studies. (DOCX) [file pone.0242403.s005.docx]

**S2 File: Summary of finding table**

|  | | | | | | |
| --- | --- | --- | --- | --- | --- | --- |
| 1. **Face mask intervention compared to no intervention for management of outbreak disease** | | | | | | |
| **Population**: Community  **Setting**: China  **Intervention**: Face mask intervention  **Comparison**: no intervention | | | | | | |
| **Outcomes** | **Anticipated absolute effects^*^ (95% CI)** | | **Relative effect (95% CI)** | **№ of participants  (studies)** | **Certainty of the evidence (GRADE)** | **Narrative result** |
|  | **Risk with no intervention** | **Risk with Face mask intervention** |  |  |  |  |
| Management of outbreak - SARS | 458 per 1,000 | **431 per 1,000** (115 to 1,000) | **RR 0.94** (0.25 to 3.57) | 1365 (2 studies) | ⊕⊕⊕⊝  Moderate ^a^ | Wearing masks lead to preventing the spread of SARS viruses among the general population |
| Management of outbreak - Influenza | 96 per 1,000 | **67 per 1,000** (31 to 145) | **RR 0.70** (0.32 to 1.51) | 844 (3 studies) | ⊕⊕⊕⊝  Moderate ^b^ | Wearing masks lead to preventing the spread of influenza viruses among the general population |
| ***The risk in the intervention group** (and its 95% confidence interval) is based on the assumed risk in the comparison group and the **relative effect** of the intervention (and its 95% CI).   **CI:** Confidence interval; **RR:** Risk ratio | | | | | | |
| **GRADE Working Group grades of evidence** **High certainty:** We are very confident that the true effect lies close to that of the estimate of the effect **Moderate certainty:** We are moderately confident in the effect estimate: The true effect is likely to be close to the estimate of the effect, but there is a possibility that it is substantially different **Low certainty:** Our confidence in the effect estimate is limited: The true effect may be substantially different from the estimate of the effect **Very low certainty:** We have very little confidence in the effect estimate: The true effect is likely to be substantially different from the estimate of effect | | | | | | |

**NB**: ^a^ Downgraded one level for imprecision of findings with a wide confidence interval.

^b^ Downgraded one level for high risk of bias in the included study.

|  | | | | | | |
| --- | --- | --- | --- | --- | --- | --- |
| 1. **Hand hygiene intervention compared to no intervention for management of outbreak disease** | | | | | | |
| **Population**: Community  **Setting**: China (3), Bangladesh (2), Thailand  **Intervention**: Hand hygiene intervention  **Comparison**: No intervention | | | | | | |
| **Outcomes** | **Anticipated absolute effects^*^ (95% CI)** | | **Relative effect (95% CI)** | **№ of participants  (studies)** | **Certainty of the evidence (GRADE)** | **Narrative result** |
|  | **Risk with no intervention** | **Risk with hand hygiene intervention** |  |  |  |  |
| Management of outbreak - SARS | 332 per 1,000 | **186 per 1,000** (27 to 1,000) | **RR 0.56** (0.08 to 3.73) | 1365 (2 studies) | ⊕⊕⊝⊝  Low ^c^ | Hand hygiene practices reduces the risk of contracting SARS among the general population |
| Management of outbreak - Influenza | 114 per 1,000 | **113 per 1,000** (106 to 119) | **RR 0.99** (0.93 to 1.04) | 1746 (4 studies) | ⊕⊕⊕⊝  Moderate ^d^ | Hand hygiene practices reduces slightly the risk of contracting Influenza among the general population |
| ***The risk in the intervention group** (and its 95% confidence interval) is based on the assumed risk in the comparison group and the **relative effect** of the intervention (and its 95% CI).   **CI:** Confidence interval; **RR:** Risk ratio | | | | | | |

**NB**: ^C^ Downgraded two levels for imprecision of findings with a wide confidence interval and high risk of bias.

^d^ Downgraded one level for high risk of bias in the included study.

|  | | | | | | |
| --- | --- | --- | --- | --- | --- | --- |
| 1. **Multi component intervention: Face mask & hand hygiene intervention compared to hand hygiene only for management of outbreak disease** | | | | | | |
| **Population**: Community  **Setting**: China, Thailand  **Intervention**: Face mask & hand hygiene intervention  **Comparison**: Hand hygiene only | | | | | | |
| **Outcomes** | **Anticipated absolute effects^*^ (95% CI)** | | **Relative effect (95% CI)** | **№ of participants  (studies)** | **Certainty of the evidence (GRADE)** | **Narrative result** |
|  | **Risk with hand hygiene only**  **intervention** | **Risk with face mask & hand hygiene intervention** |  |  |  |  |
| Management of outbreak - influenza | 120 per 1,000 | **131 per 1,000** (94 to 181) | **RR 1.09** (0.78 to 1.50) | 906 (2 studies) | ⊕⊕⊕⊕  High ^e^ | Combined intervention of face masks and hand hygiene did not show improvement of preventing the spread of influenza among the general population |
| ***The risk in the intervention group** (and its 95% confidence interval) is based on the assumed risk in the comparison group and the **relative effect** of the intervention (and its 95% CI).   **CI:** Confidence interval; **RR:** Risk ratio | | | | | | |
| **GRADE Working Group grades of evidence** **High certainty:** We are very confident that the true effect lies close to that of the estimate of the effect **Moderate certainty:** We are moderately confident in the effect estimate: The true effect is likely to be close to the estimate of the effect, but there is a possibility that it is substantially different **Low certainty:** Our confidence in the effect estimate is limited: The true effect may be substantially different from the estimate of the effect **Very low certainty:** We have very little confidence in the effect estimate: The true effect is likely to be substantially different from the estimate of effect | | | | | | |

**NB**: ^e^ Downgraded one level due to unclear risk of bias in the studies.

|  | | | | | | |
| --- | --- | --- | --- | --- | --- | --- |
| 1. **Multi component intervention: Face mask & hand hygiene intervention compared to no intervention for management of outbreak disease** | | | | | | |
| **Population**: Community  **Setting**: China, Thailand  **Intervention**: Face mask & hand hygiene intervention  **Comparison**: No intervention | | | | | | |
| **Outcomes** | **Anticipated absolute effects^*^ (95% CI)** | | **Relative effect (95% CI)** | **№ of participants  (studies)** | **Certainty of the evidence (GRADE)** | **Narrative result** |
|  | **Risk with no**  **intervention** | **Risk with face mask & hand hygiene intervention** |  |  |  |  |
| Management of outbreak - influenza | 154 per 1,000 | **145 per 1,000** (89 to 237) | **RR 0.94** (0.58 to 1.54) | 923 (2 studies) | ⊕⊕⊕⊕  High ^f^ | Combined intervention of face masks and hand hygiene improved the spread of influenza among the general population |
| ***The risk in the intervention group** (and its 95% confidence interval) is based on the assumed risk in the comparison group and the **relative effect** of the intervention (and its 95% CI).   **CI:** Confidence interval; **RR:** Risk ratio | | | | | | |
| **GRADE Working Group grades of evidence** **High certainty:** We are very confident that the true effect lies close to that of the estimate of the effect **Moderate certainty:** We are moderately confident in the effect estimate: The true effect is likely to be close to the estimate of the effect, but there is a possibility that it is substantially different **Low certainty:** Our confidence in the effect estimate is limited: The true effect may be substantially different from the estimate of the effect **Very low certainty:** We have very little confidence in the effect estimate: The true effect is likely to be substantially different from the estimate of effect | | | | | | |

**NB**: ^f^ Downgraded one level due to unclear risk of bias in the studies.

|  | | | |
| --- | --- | --- | --- |
| 1. **Social intervention compared to no intervention for management of outbreak disease** | | | |
| **Population**: Community  **Setting**: Romania, Serbia, Thailand, Madagascar, Mexico, Peru (2), Mexico (2)  **Intervention**: Social intervention  **Comparison**: No intervention | | | |
| **Outcomes** | **№ of participants  (studies)** | **Certainty of the evidence (GRADE)** | **Narrative result** |
| Captured opportunities for social distancing | > 200,000 (9 studies) | ⊕⊝⊝⊝  Very low ^g^ | Minimizing gathering i.e. school closures slow down the spread of influenza |
| ***The risk in the intervention group** (and its 95% confidence interval) is based on the assumed risk in the comparison group and the **relative effect** of the intervention (and its 95% CI).   **CI:** Confidence interval; **RR:** Risk ratio | | | |
| **GRADE Working Group grades of evidence** **High certainty:** We are very confident that the true effect lies close to that of the estimate of the effect **Moderate certainty:** We are moderately confident in the effect estimate: The true effect is likely to be close to the estimate of the effect, but there is a possibility that it is substantially different **Low certainty:** Our confidence in the effect estimate is limited: The true effect may be substantially different from the estimate of the effect **Very low certainty:** We have very little confidence in the effect estimate: The true effect is likely to be substantially different from the estimate of effect | | | |

**NB**: ^g^ Downgraded three levels for imprecision of findings, unclear risk of bias & high risk of bias in the included study
